# Inter-Stage Output Voltage Amplitude Improvement Circuit Integrated with Class-B Transmit Voltage Amplifier for Mobile Ultrasound Machines

**DOI:** 10.3390/s20216244

**Published:** 2020-11-02

**Authors:** Kiheum You, Hojong Choi

**Affiliations:** Department of Medical IT Convergence Engineering, Kumoh National Institute of Technology, Gumi 39253, Korea; rlgma12@kumoh.ac.kr

**Keywords:** inter-stage, output voltage amplitude, transmit voltage amplifier, mobile ultrasound machine, piezoelectric transducer

## Abstract

Piezoelectric transducers are triggered by the output voltage signal of a transmit voltage amplifier (TVA). In mobile ultrasound instruments, the sensitivity of piezoelectric transducers is a critical parameter under limited power supply from portable batteries. Therefore, the enhancement of the output voltage amplitude of the amplifier under limited power supply could increase the sensitivity of the piezoelectric transducer. Several-stage TVAs are used to increase the voltage amplitude. However, inter-stage design issues between each TVA block may reduce the voltage amplitude and bandwidth because the electronic components of the amplifier are nonlinearly operated at the desired frequency ranges. To compensate for this effect, we propose a novel inter-stage output voltage amplitude improvement (OVAI) circuit integrated with a class-B TVA circuit. We performed fundamental A-mode pulse-echo tests using a 15-MHz immersion-type piezoelectric transducer to verify the design. The echo amplitude and bandwidth when using an inter-stage OVAI circuit integrated with a class-B TVA circuit (696 mV_PP_ and 29.91%, respectively) were higher than those obtained when using only the class-B TVA circuit (576 mV_PP_ and 24.21%, respectively). Therefore, the proposed OVAI circuit could be beneficial for increasing the output amplitude of the class-B TVA circuit for mobile ultrasound machines.

## 1. Introduction

Piezoelectric transducers are used to generate very weak acoustic waves or electrical signals as energy sources owing to high signal loss [1,2,3]. Ultrasound machines are critical sensor devices because of the very high electro-mechanical conversion loss caused by the characteristics of piezoelectric materials [4,5,6,7,8]. Compared with the piezoelectric transducers used in desktop ultrasound machines, the piezoelectric transducers used in mobile ultrasound machines suffer from a lower sensitivity due to the smaller size of the piezoelectric material resulting from the limited portable battery and closed structures of these machines. Smaller-sized materials need to be utilized to fabricate piezoelectric transducers in order to obtain stable performances due to heat generation. Under a limited battery power, a higher voltage generation from transmit voltage amplifier (TVA) devices is highly desirable [9,10,11]. 

A several-stage TVA must be constructed to increase the voltage generation in order to improve the sensitivity of piezoelectric transducers. When the amplifier devices are connected in series, several electrical performance issues, such as input/output impedances, maximum voltage swings, and current dissipation, occur [12,13,14]. These issues directly affect the performance of the ultrasound components. 

Mobile ultrasound machines have several electrical and physical parameters, such as sensitivity, resolution, contrast ratio, battery capability, heat transfer, size, and weight [9,15]. The sensitivity, resolution and contrast ratio of mobile ultrasound machines are directly related to the voltage amplitude and bandwidth of the piezoelectric transducer, the TVA in the transmitter, and the analog-to-digital converter (ADC) in the receiver. The battery capability and heat transfer of mobile ultrasound machines are related to the current consumption of the TVA in the transmitter and the ADC in the receiver [16]. The size, cooling system, and weight of mobile ultrasound machines are also related to the performances of the transducer and system because the limited size and weight of mobile ultrasound machines require the use of a small-sized cooling system; thus, heat generation limits the performance of the ultrasound components [10].

High voltage generation from the TVA causes unwanted heat transfer in the aluminum cases of mobile ultrasound instruments. Therefore, mobile ultrasound companies unfortunately lower the voltage amplitude generation from the TVA for circuit protection and system stability [9,17]. Several-stage TVAs need to be constructed to increase the voltage amplitude generation. However, there are interaction issues between consecutive amplifiers because the electronic components in each amplifier are operated nonlinearly [18]. This creates multi-dimensional design problems for the TVA in mobile ultrasound machines. As described previously, several parameter tradeoffs present challenging design issues to construct high-performance amplifiers, thus compromising the sensitivity performance of the piezoelectric transducers for mobile ultrasound machines. 

As the size of mobile ultrasound instruments is limited, multiplexer and de-multiplexer circuits with small numbers of TVAs must be used [9]. Therefore, a high output voltage amplitude generated from only one TVA is more desirable for mobile ultrasound machines compared with desktop ultrasound machines. 

Different types of amplifiers, such as class-A, -B, -C, and -D type amplifiers, have been proposed [19,20,21,22]. Class-A type amplifiers can generate a very high voltage with a high linearity but require very high current consumption [23,24]. Therefore, they are not suitable for mobile ultrasound machines because of portable battery life. However, the commercial amplifiers currently used in mobile ultrasound machines are class-A type amplifiers [25,26,27]. Compared with class-A type amplifiers, class-B amplifiers can generate a relatively lower voltage with lower linearity and current consumption [28,29]. However, they have a higher voltage generation capability and higher linearity with higher current consumption compared with class-C or -D type amplifiers [30,31]. 

Several studies have been conducted to improve the TVA used in ultrasound machines. Texas Instruments attempted to use a pre-distortion technique by reducing unwanted harmonic distortions, thus improving the image quality of ultrasound machines [19]. Texas Instruments utilized the ADC and digital-to-analog converter (DAC) with memory in the field programming gate array (FPGA) board such that this technique is useful in reducing the harmonic components [19]. However, the gain and bandwidth of the amplifier cannot be adjusted. In addition, this approach may not be suitable for mobile ultrasound systems because ADC and DAC with memory leads to higher power consumption and greater size such that it is very difficult to be applied to the array type transducer. [18]. A matching filter with an amplifier was developed to increase the bandwidths of the piezoelectric transducer [32]. Using the impedance matching technique, the technique is simple, so it is useful for implementation in the array type transducer [31]. The bandwidth of the amplifier with ultrasonic transducer could be adjusted. However, voltage gain decreases because this approach utilizes only passive components. Accordingly, the amplitude of the echo signal decreases.

In previous research, the post-voltage-boost circuit was intended to control the output impedances of the second-stage amplifier and the analog filter and the piezoelectric transducer together to increase the amplitude and reject unwanted harmonics regardless of the expander and filter performances [33]. However, the current consumption of the amplifier shows a greater increase with the help of the post-voltage-boost circuit. Therefore, it is trade-off between gain and current consumption.

On the other hand, this proposed inter-stage output amplitude improvement (OVAI) circuit approach aims to control the output impedance of the first-stage amplifier and input impedance of the second-stage amplifier to overcome the inter-connection amplifier problems, such as the voltage amplitude and bandwidth of the amplifier itself. In constructing the multi-stage amplifier to increase the voltage amplitude, there are inter-connection issues. The varied internal capacitance and resistance values in the OVAI circuit could reduce the effects on the non-linear capacitances and inductances in the output and input ports of first- and second-stage amplifiers. In addition, inter-stage design affects the expander and filter performances a little, because the proposed design is located between the amplifiers if the piezoelectric transducer is stable for impedance variances.

**Figure 1:**
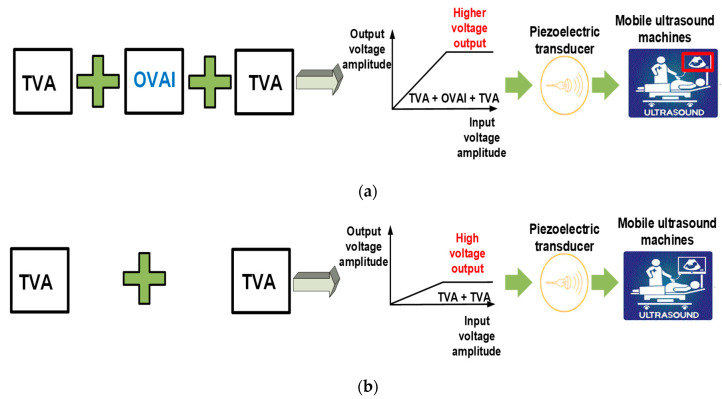
Concept of the developed transmit voltage amplifier (TVA) circuit (**a**) with and (**b**) without the output voltage amplitude improvement (OVAI) circuit for mobile ultrasound machines.

Furthermore, the OVAI circuit could improve the gain and bandwidth. However, this approach does not influence current consumption through the shunt capacitor and shunt diode of the OVAI circuit. Therefore, this approach could be useful for a mobile ultrasound system which has limited current consumption due to portable batteries. To the best of our knowledge, we are the first to propose a design for an inter-stage OVAI circuit for ultrasound applications, since the multiple-stage TVA circuit used for ultrasound transducer research has inter-connection issues. Accordingly, we enhance the voltage amplitude generated from a TVA circuit with a relatively low current consumption for mobile ultrasound instruments. In ultrasound systems, matching filters are usually located between the TVA circuit and the piezoelectric transducer. However, the proposed OVAI circuit will be located between the TVA circuits; hence, it can reduce interaction issues with the filters and transducers. Figure 1 illustrates the concept of the proposed OVAI circuit, with a TVA circuit and piezoelectric transducer for mobile ultrasound machines. With a designed OVAI circuit, a higher voltage output amplitude generated from the TVA can be applied to the piezoelectric transducers. Thus, a higher sensitivity of the piezoelectric transducers may be obtained. 

The remainder of this article is organized as follows. Section 2 describes the design and mathematical circuit analysis of the inter-stage OVAI circuit integrated with a class-B TVA circuit. Section 3 presents the test setup and measurement results of the TVA circuit with and without the OVAI circuit, including the A-mode pulse-echo test result using a piezoelectric transducer. Section 4 concludes the paper.

## 2. Materials and Methods

**Figure 2:**
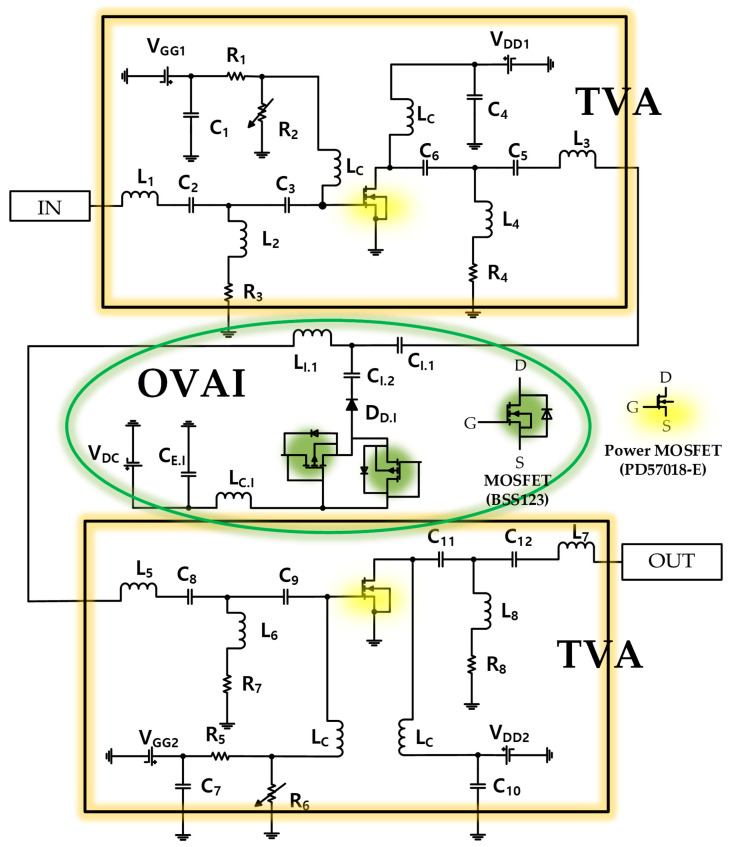
Schematics of the proposed TVA with the OVAI circuit.

The OVAI circuit is located between the first- and second-stage TVAs. The output voltage generated by the first-stage TVA is transmitted to the second-stage TVA through the OVAI circuit. Therefore, the OVAI circuit is designed to produce a higher output voltage applied to the piezoelectric transducer, thus generating a higher ultrasonic signal. Here, the circuit configurations of the first-and second-stage TVAs are the same. The schematics of the TVA circuit integrated with the OVAI circuit are shown in Figure 2.

As shown, the primary transistor (power metal–oxide–semiconductor field-effect transistor (MOSFET), STMicroelectronics, PD57018-E) was used to drive the TVA in the class-B mode. A radio frequency (RF) choke inductor (L_C_, 1 µH) was used to minimize the voltage drop when a DC voltage was applied [34]. In addition, electrolytic capacitors (C_1_, C_4_, C_7_, C_10_ = 220 µF) were used to minimize the noise signal from the DC power supply [35]. The input (L_1_ = 22 nH, C_2_ = 560 pF, C_3_ = 330 pF, L_2_ = 1000 nH, R_3_ = 200 Ω) and output (L_3_ = 120 nH, C_5_ = 330 pF, C_6_ = 820 pF, L_4_ = 500 nH, R_4_ = 200 Ω) ports of the first- and second-stage TVA circuits were configured for 50-Ω impedance matching the center frequency of 15 MHz.

### 2.1. Equivalent Circuit Model of TVA and OVAI Circuits for DC Analysis

**Figure 3:**
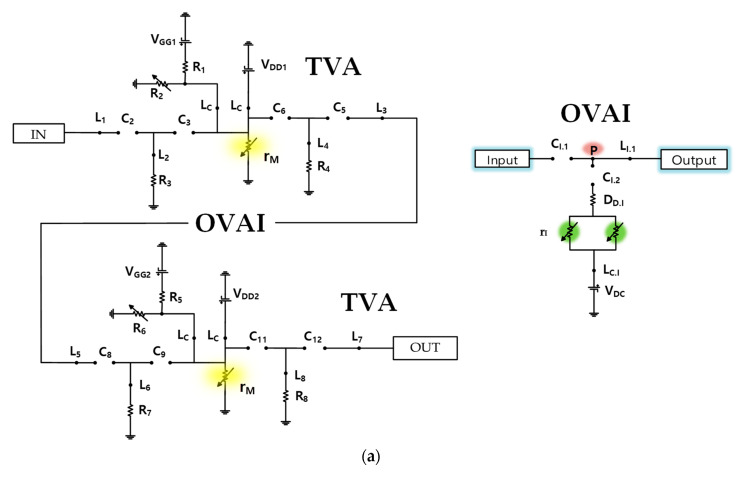

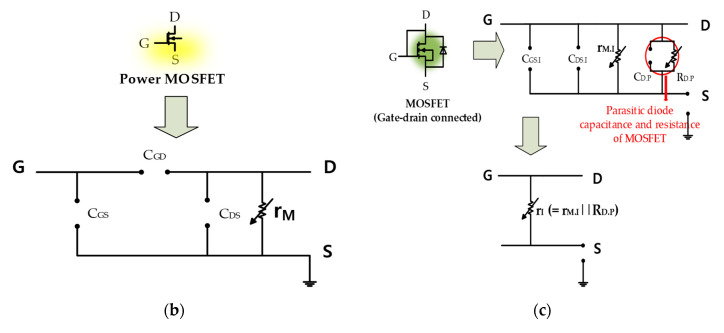
(**a**) Equivalent circuit model of the TVA circuit and OVAI circuit; (**b**) power metal-oxide-semiconductor field-effect transistor (MOSFET) and (**c**) MOSFET for DC analysis.

This section investigates the battery effect of the TVA with the OVAI circuits in a mobile ultrasound system through an equivalent circuit model for DC analysis. Figure 3a shows the DC analysis of the TVA+OVAI+TVA circuit (first- and second-stage TVAs with the OVAI circuit). In the ideal case of the DC analysis, the capacitor (1/jwC = ∞) is open and the inductor (jwL = 0) is short in the TVA and OVAI circuit. The equivalent model of the diode in the OVAI circuit consists of a parallel connection of a small variable resistor (D_D.I_) and an open capacitor. In the TVA circuit, the internal parasitic gate-source, gate-drain, and drain-source capacitors (C_GS_, C_GD_, and C_DS_, respectively) of the power MOSFET become open. The power MOSFET has a variable drain-source resistor (r_M_), as shown in Figure 3b. In the OVAI circuit, the MOSFET’s internal capacitor to which the gate drain is connected becomes open. However, the source of the MOSFET is not grounded; hence, the internal variable resistor (r_I_) has a very large value. r_I_ is the resistance combination of r_M.I_ and R_D.P_ and is shown in Figure 3c. Owing to the large value of the internal resistor (r_I_), the current consumption could be assumed to have a small value. Owing to the open circuit of C_I.2_, the effect of the internal resistor of the MOSFET is small, and the DC applied current does not reach point P; consequently, its effect is small. Finally, the OVAI circuit in DC mode has a slight effect on the current consumption; hence, the OVAI circuit has a slight DC current effect on the TVA+OVAI+TVA circuit.

### 2.2. Equivalent Circuit Model of TVA and OVAI Circuits for AC Analysis

The internal capacitance of the MOSFET (BSS123) of the OVAI circuit varies considerably in the capacitors (C_GS.I_ and C_DS.I_) according to the DC bias (V_DC_) applied to the drain of the MOSFET. This capacitance (C_M.I_) also helps compensate for the impedances Z_out1_ of the first-stage TVA and Z_in2_ of the second-stage TVA circuits. 

**Figure 4:**
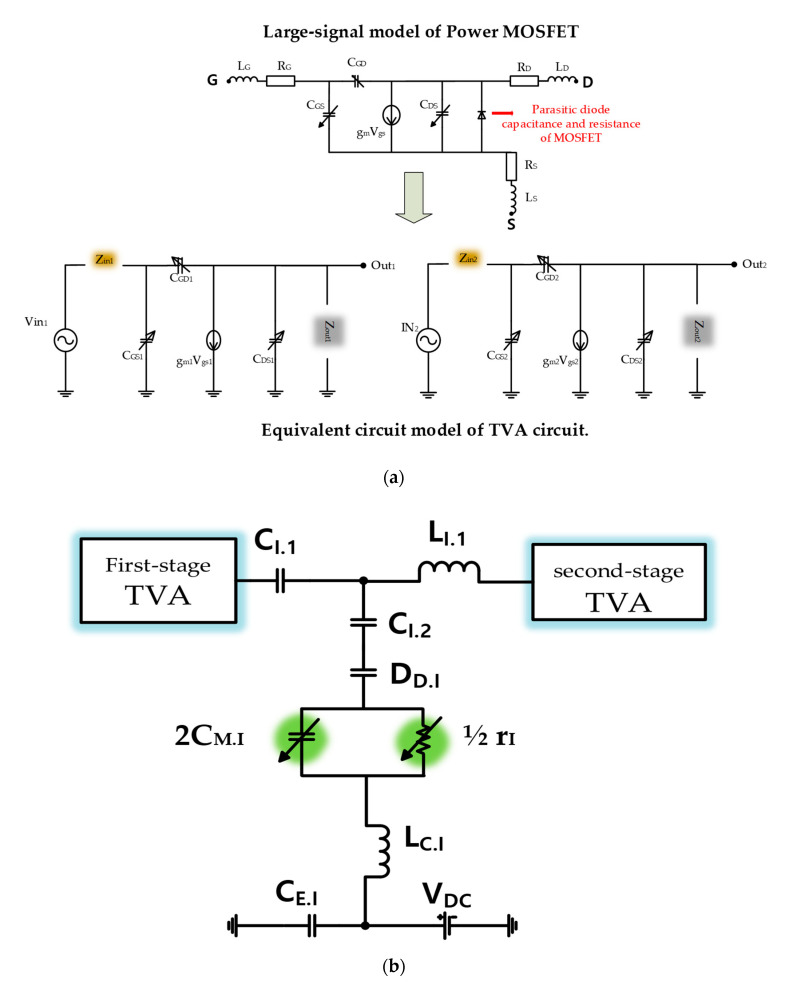

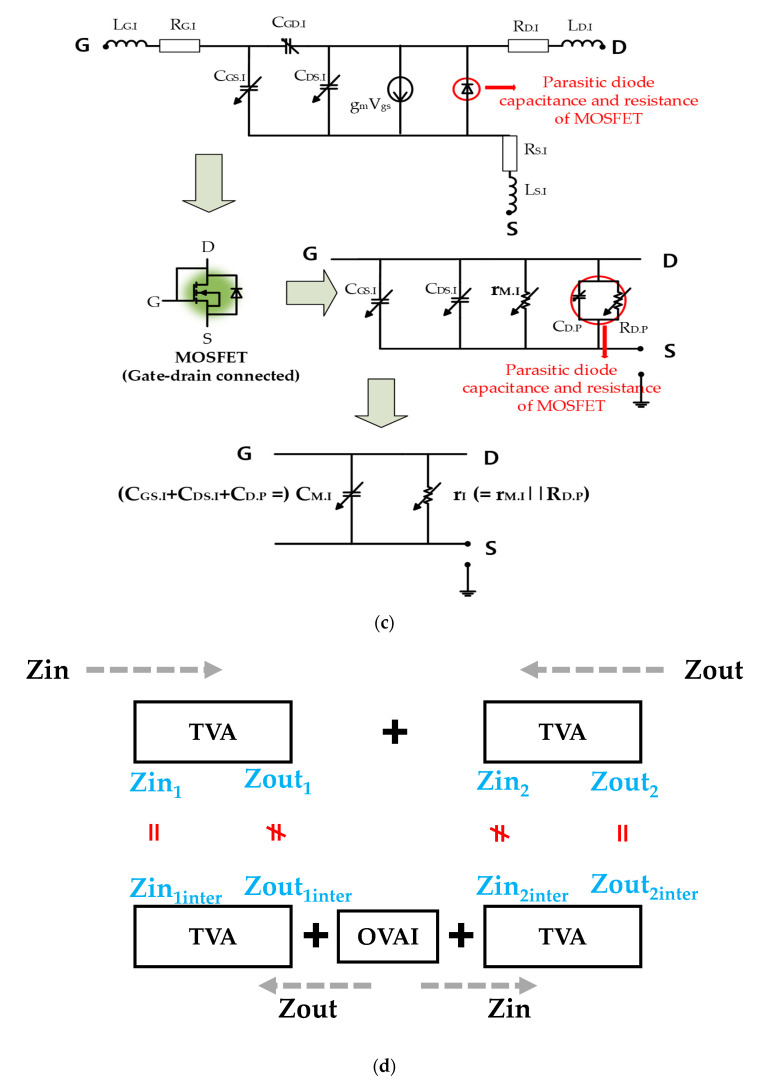
(**a**) Equivalent circuit model of the first- and second-stage TVA circuits and (**b**) OVAI circuits and (**c**) MOSFET for AC analysis; (**d**) Impedance model concept of the OVAI circuit between the first- and second-stage TVA circuits.

Figure 4 provides an equivalent circuit diagram of the TVA with OVAI circuits for AC analysis. Mathematical analysis with a large-signal transistor equivalent circuit model will be performed to guide the design of the TVA circuit integrated with the OVAI circuit. Figure 4a shows the large-signal equivalent circuit model of the power MOSFET (PD57018-E) [28,36]. The L_G_ and R_G_ in the large-signal model were added to Z_in1_ and Z_in2_ of the TVA circuit equivalent model, and the R_D_, L_D_, R_S_, and L_S_ in the large-signal model were also added to Z_out1_ and Z_out2_. The C_GS_, C_GD_, C_DS_, and g_m_ are the internal capacitors of the transistors and transconductance, respectively. The C_DS1_ and C_DS2_ of the TVA circuit equivalent model are the sum of C_DS_ and the parasitic diode of MOSFET of the large-signal model. Figure 4b shows the equivalent circuit of the OVAI. The diode in the OVAI circuit consists of a small-value variable resistor and a capacitor (D_D.I_) connected in parallel, and the small-value resistor is neglected. In Figure 4c, we used the original large-signal transistor equivalent circuit model for AC analysis as below. Our application operated at 15 MHz such that we can simplify the original large-signal transistor equivalent circuit model by reducing some parasitic inductances and resistances (L_G.I_, L_D.I_, L_S.I_, and R_G.I_, R_S.I_, R_D.I_). The transistor (BSS123) used in the OVAI circuit is gate-drain connected; hence, it operates as a variable capacitor (C_M.I_) in the equivalent circuit model. Figure 4d is provided to help understand the impedance concept for the TVA with OVAI circuits.

The following mathematical analysis illustrates the effect of the OVAI circuit on the TVA. Z_in1_ is the impedance seen from the input side in the first-stage TVA circuit. The Z_in1_ of the TVA+TVA (first- and second-stage TVAs only) circuit and the Z_in1inter_ of the TVA+OVAI+TVA circuit are the same (Z_in1_ = Z_in1inter_).

(1) $$Zin_{1}=\;\;Zin{\;}_{1\mathrm{inter}}\;=\;\;(sL_{1}+\frac{1}{sC_{2}})+\left\{(sL_{2}+R_{3})∥\frac{1}{sC_{3}}\right\}$$

where s is *j2πf_c_*, and *f_c_* is the center frequency. In Z_in1_, the series-connected L_1_ and C_2_ are parallel to L_2_, R_3_, and C_3_. We used s instead of *f_c_* to simplify the equations.

Z_out1_ is the impedance seen from the output side in the first-stage TVA circuit. Here, Z_out1inter_, which is the impedance including the input side of the OVAI circuit, and Z_out1_, which does not include the input side of the OVAI circuit, are different. First, the Z_out1_ of the TVA+TVA circuit is expressed as follows. On the output side of the first-stage TVA circuit, the series-connected L_3_ and C_5_ are parallel to L_4_ and R_4_, and C_6_.

(2) $$Zout_{1}=\;(sL_{3}+\frac{1}{sC_{5}})+\left\{(sL_{4}+R_{4})∥\frac{1}{sC_{6}}\right\}$$

The Z_out1inter_ of the TVA+OVAI+TVA circuit is the impedance seen from the OVAI circuit toward the output side of the first-stage TVA circuit. The OVAI circuit between the first- and second-stage TVA circuits affects the Z_out1inter_ of the first-stage TVA circuit. Finally, the output of the OVAI circuit is delivered to the input of the second-stage TVA circuit. The Z_out1inter_ of the TVA+OVAI+TVA circuit is expressed as follows: Z_out1_ is composed of the choke inductor L_C.I_ in the OVAI circuit, the variable capacitor C_M.I_ of the transistor, the capacitor C_D.I_ of the diode, and the series-connected capacitors C_I.2_ and C_I.1_, L_3_, and C_5_. It is also parallel to L_4_ and R_4_, and C_6_.

(3) $$Zout_{1\mathrm{inter}}=\;\;\{sL_{C.I}+(\frac{1}{2sC_{M.I}}∥\frac{r_{I}}{2})+\frac{1}{sC_{D.I}}+\frac{1}{sC_{I.2}}\}∥(\frac{1}{sC_{I.1}}+sL_{3}+\frac{1}{sC_{5}})+(sL_{4}+R_{4})∥\frac{1}{sC_{6}}$$

where C_M.I_ is a variable capacitor connected in parallel with the internal variable capacitance (C_GS.I_ and C_DS.I_) and the capacitance (C_D_) of the parasitic diode of the MOSFET (BSS123). r_I_ is a variable resistor connected in parallel between the internal small resistance and the resistance of the parasitic diode of the MOSFET (BSS123). Z_out1_ is composed of the choke inductor L_C.I_ of the OVAI circuit, the variable capacitor C_M.I_ and variable resistor (r_I_) of the transistor, the capacitor C_D.I_ of the diode, and the capacitors C_I.2_ and C_I.1_, L_3_, and C_5_. It is also parallel to L_4_ and R_4_, and C_6_. Therefore, the capacitances and inductances of the OVAI circuit could compensate for the inductance of the output impedance of the first-stage TVA circuit.

Z_in2_ is the impedance seen from the input side of the second-stage TVA circuit. The Z_in2inter_ with the OVAI circuit and the Z_in2_ without the OVAI circuit are different. First, the Z_in2_ of the TVA+TVA circuit is expressed as follows. Z_in2_ is composed of the series-connected L_5_ and C_8_ in parallel with L_6_ and R_7_, and C_9_.

(4) $$Zin_{2}=\;(sL_{5}+\frac{1}{sC_{8}})+\left\{(sL_{6}+R_{7})∥\frac{1}{sC_{9}}\right\}.$$

The Z_in2inter_ of the TVA+OVAI+TVA circuit is the impedance seen from the output side of the OVAI circuit toward the input side of the second-stage TVA circuit. The OVAI circuit between the first- and second-stage TVA circuits affects Z_out1inter_ in the first stage, which affects Z_in2inter_ in the second stage. Finally, the signal passing through the first-stage TVA and OVAI is affected by the input of the second stage. The Z_in2inter_ of the TVA+OVAI+TVA circuit is expressed as follows: Z_in2inter_ is composed of the series-connected L_I.1_, L_5_, and C_8_ of the OVAI circuit connected in parallel with L_6_ and R_7_, and then connected in series with C_9_. Therefore, the inductance of the OVAI circuit could compensate for the capacitance of the input impedance of the second-stage TVA circuit.

(5) $$Zin_{2\mathrm{inter}}=\;(sL_{I.1}+sL_{5}+\frac{1}{sC_{8}})+(sL_{6}+R_{7})∥\frac{1}{sC_{9}}.$$

Z_out2_ is the impedance seen from the output side of the second-stage TVA circuit. The Z_out2_ of the TVA+TVA circuit and the Z_out2inter_ of the TVA+OVAI+TVA circuit are the same (Z_out2_ = Z_out2inter_). Z_out2_ is composed of the series-connected L_7_ and C_12_ in parallel to L_8_, R_8_, and C_11_.

(6) $$Zout_{2}=\;\;Zout_{2\mathrm{inter}}\;=\;\;(sL_{7}+\frac{1}{sC_{12}})+\left\{(sL_{8}+R_{8})∥\frac{1}{sC_{11}}\right\}.$$

The pole *w* of the TVA+TVA circuit was obtained from Equations (1), (2), (4) and (6). *w*_in1_ and *w*_in2_ are the input poles of the first- and second-stage TVA circuits, respectively.

(7) $$\omega_{in1}=\frac{1}{Zin_{1}[C_{GS1}+(1+g_{m1}Zout_{1})C_{GD1}]},\;\;\;\omega_{in2}=\frac{1}{Zin_{2}[C_{GS2}+(1+g_{m2}Zout_{2})C_{GD2}]}.$$

The pole *w* of the TVA+OVAI+TVA circuit is affected by Z_outinter_. The input pole *w*_ininter_ of the TVA+OVAI+TVA circuit is affected by the impedance of the OVAI circuit from Equations (1), (3), (5) and (6). *w*_in1inter_ and *w*_in__2inter_ are the input poles of the first- and second-stage of the TVA+OVAI+TVA circuit.

(8) $$\omega_{in1\mathrm{inter}}=\frac{1}{Zin_{1}[C_{GS1}+(1+g_{m1}Zout_{1\mathrm{inter}})C_{GD1}]},\;\omega_{in2\mathrm{inter}}=\frac{1}{Zin_{2\mathrm{inter}}[C_{GS2}+(1+g_{m2}Zout_{2\mathrm{inter}})C_{GD2}]}.$$

The output poles *w* of the TVA+TVA circuit or TVA+OVAI+TVA circuit was obtained from Equations (1), (2), (4) and (6). *w*_out1_ and *w*_out2_ are the output poles of the first- and second-stage TVA+TVA circuits, respectively.

(9) $$\omega_{out1}=\frac{1}{Zout_{1}(C_{DB1}+C_{GD1})},\;\;\;\;\omega_{out2}=\frac{1}{Zout_{2}(C_{DB2}+C_{GD2})}.$$

The output poles *w* of the TVA+OVAI+TVA circuit are affected by Z_out2nter_. The output pole *w*_outinter_ of the TVA+OVAI+TVA circuit is affected by the impedance of the OVAI circuit from Equations (1), (3), (5), and (6). *w*_out1inter_ and *w*_out2inter_ are the output poles of the first- and second-stage TVA+OVAI+TVA circuits, respectively.

(10) $$\omega_{out1\mathrm{inter}}=\frac{1}{Zout_{1\mathrm{inter}}(C_{DB1}+C_{GD1})},\;\;\omega_{out2\mathrm{inter}}=\frac{1}{Zout_{2\mathrm{inter}}(C_{DB2}+C_{GD2})}.$$

Through Equations (7) and (9), the output values of the TVA+TVA circuit can be obtained as follows: Out_1_ and Out_2_ are the outputs of the first- and second-stage TVA+TVA circuits, respectively.

(11) $$Out_{1}(s)=\frac{-g_{m1}Zout_{1}}{\left(1+\frac{s}{\omega_{in1}})(1+\frac{s}{\omega_{out1}}\right)}*Vin,\;\;\;Out_{2}(s)=\frac{-g_{m2}Zout_{2}}{\left(1+\frac{s}{\omega_{in2}})(1+\frac{s}{\omega_{out2}}\right)}*Vin.$$

Through Equations (8) and (10), the output values of the TVA+OVAI+TVA circuit can be obtained as follows: Out_1inter_ and Out_2inter_ are the outputs of the first- and second-stage TVA+TVA circuits, respectively.

(12) $$Out_{1\mathrm{inter}}(s)=\frac{-g_{m1}Zout_{1}}{\left(1+\frac{s}{\omega_{in1\mathrm{inter}}})(1+\frac{s}{\omega_{out1\mathrm{inter}}}\right)}*Vin,\;\;\;Out_{2\mathrm{inter}}(s)=\frac{-g_{m2}Zout_{2\mathrm{inter}}}{\left(1+\frac{s}{\omega_{in2\mathrm{int}er}})(1+\frac{s}{\omega_{out2\mathrm{inter}}}\right)}*Vin,$$

where the V_in_ of the TVA+TVA circuit and the V_in_ of the TVA+OVAI+TVA circuit are equal.

In Equation (12), the compensated input and output poles could reduce the nonlinear capacitances and inductances, thus increasing the output voltage amplifier. The final output can be obtained by multiplying the outputs from the first- and second-stage TVA circuits. With Equations (11) and (12), it is impossible to analyze the effects of the nonlinear capacitances and inductance on the input and output poles because several capacitance and inductance variables in the TVA and OVAI circuits interact with each other. However, from Equations (11) and (12), the final outputs of the two-stage TVA+TVA (V_OUT_) and TVA+OVAI+TVA (V_OUTinter_) circuits were obtained.

(13) $$V_{OUT}=Out_{1}*\frac{Out_{2}}{V_{in2}}\;\;\;\;\;\neq \;\;\;\;\;V_{OUTinter}=Out_{1\mathrm{inter}}*\frac{Out_{2\mathrm{int}er}}{V_{in2linear}}.$$

where V_in2_ is the input voltage of the second-stage TVA circuit and V_in2linear_ is the input voltage of the OVAI circuit.

According to the datasheets of the MOSFET (BSS123) used in the OVAI circuit and the electrical characteristics of MOSFETs, the parasitic input capacitance (C_ISS_) is the combination of the gate-source (C_GS.I_) and gate-drain (C_GD.I_) capacitances, parasitic output capacitance (C_OSS_) is the combination of the gate-drain (C_GD.I_) and drain-source (C_DS.I_) capacitances, and the reverse capacitance (C_RSS_) is the gate-drain capacitance (C_GD.I_) [37]. The value changes in the parasitic input, output, and reverse capacitances (C_ISS_, C_OSS_, and C_RSS_) of the power MOSFET according to the drain voltage of the MOSFET can be dramatically varied as shown in the datasheet [37]. The DC bias voltage of the OVAI circuit with 0.5, 1, and 2 V were applied to adjust the gain and bandwidth through the dramatic changes of C_ISS_, C_OSS_, and C_RSS_ of the power MOSFET (BSS123). Therefore, we can conclude that the capacitance values at the drain voltage less than 10 V, the C_ISS_, C_OSS_, and C_RSS_ decreased rapidly.

In Equation (3), the capacitance component (C_M.I_, C_D.I_, C_I.2_, and C_I.1_) of the impedance Z_out1inter_ could offset the inductances (L_3_ and L_4_) of the first-stage TVA, and the inductance component (L_C.I_) could also offset the capacitance components (C_5_ and C_6_) of first-stage TVA. In Equation (5), the inductance component (L_I.1_) of Zin2inter could compensate the capacitance component (C_8_ and C_9_) of the second-stage TVA. Therefore, the impedances Z_out1inter_ and Z_in2inter_ have a smaller value than the impedances Z_out1_ and Z_in2_. The input and output poles of Equations (8) and (10) have a larger value than the input and output poles of Equations (7) and (9). For the larger pole values in the common-source stage amplifier, the bandwidth could be improved [28]. Since the input and output poles value increase, the bandwidth of the TVA circuit can be enhanced accordingly. In addition, the impedances Out_1inter_ and Out_2inte_r in Equation (12) have larger values than the impedances Out_1_ and Out_2_ in Equation (11), thus the amplitude of the TVA circuit could possibly be increased.

The power MOSFET and MOSFET used in the analysis have variable and inaccurate analytical parameters owing to the differences in environmental temperature and DC voltage levels. Therefore, there must be errors between the experimental and theoretical results in a high-voltage test environment. It is very challenging to predict the performance of the amplifier accurately owing to inaccurate signal distortion that appears in MOSFET simulation libraries under a high-voltage environment [38,39]. In addition, the output performances of the amplifier can fluctuate considerably in the experiment owing to external environmental factors, such as temperature and power level parameters [38,40]. Therefore, the theoretical and expected performances of the amplifier under a high-voltage environment are not accurate, and the performances of the amplifier (TVA) and inter-stage OVAI circuit should be verified using measurement data.

## 3. Results

**Figure 5:**
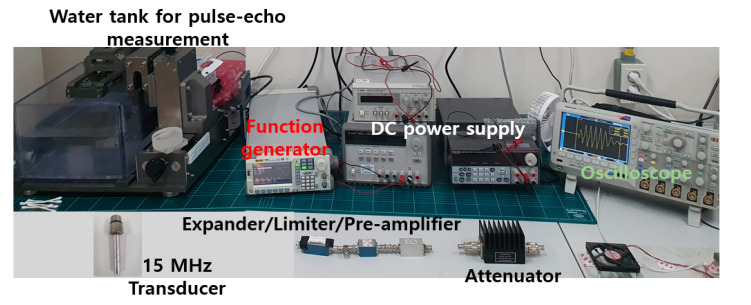
Testing environment for the developed circuit.

Figure 5 shows the experimental environment. The arbitrary function generator (DG5071, Rigol Technologies Inc., Beijing, China), DC power supply (E3630A, E3631A, and 6010A, Keysight Technologies, Santa Clara, CA, USA) and oscilloscope (MDO4104C, Tektronix Inc., Beaverton, OR, USA) were used to evaluate the output voltages and currents of the TVA+TVA and TVA+OVAI+TVA circuits. After the output voltages and currents were obtained using the oscilloscope, the gain and spectrum data were processed.

Components such as an expander, limiter, water tank, transducer, quartz, and double-distilled water were used for the pulse-echo analysis, in addition to the aforementioned instruments because the performance of the transducer needs to be measured using the developed circuits to obtain the pulse-echo response [41]. The expander used in the pulse-echo analysis consisted of a pair of diodes, which was used to reduce the ring-down signal generated from the TVA circuit [42]. In the limiter used in the pulse-echo analysis, a pair of diodes was connected in parallel with a resistor. The limiter was used to reduce the high-voltage signal generated from the TVA circuit to protect the oscilloscope and pre-amplifier. The transducer used in the pulse-echo analysis was a 15-MHz 1/4”-diameter ultrasonic immersion transducer provided by Olympus (I21504T, Shinjuku-ku, Tokyo, Japan).

**Figure 6:**
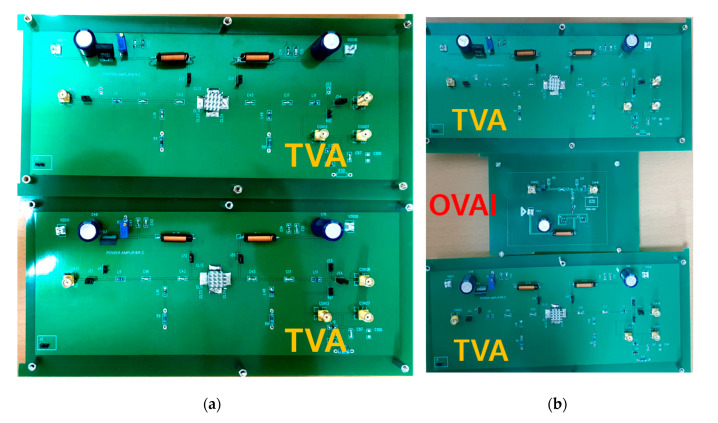
(**a**) Printed circuit board (PCB) of the TVA+TVA circuit; (**b**) PCB of the TVA+OVAI+TVA circuit.

A printed circuit board (PCB) with the TVA and OVAI circuits is shown in Figure 6. The TVA+OVAI+TVA circuit was constructed and connected through a cable. The output signal was transmitted from the first-stage TVA to the input port of the second-stage TVA through the OVAI circuit, and the output port of the second-stage TVA was connected to the oscilloscope to measure the final output.

The high-power choke inductor, electrolytic capacitors, and power resistors in the PCB must be stable under a high-voltage environment. In particular, a heat sink and external cooler are required to minimize the temperature influence on the power MOSFET, which is a temperature-sensitive electronic component, for a stable test environment [39,43].

### 3.1. Performance Measurement and Analysis of the TVA+TVA and TVA+OVAI+TVA Circuits

**Figure 7:**
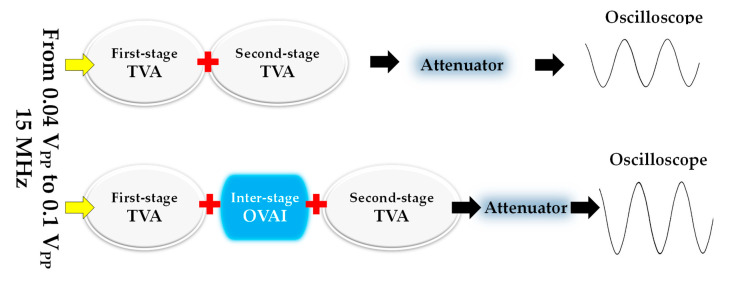
Block diagram of the measurement of the performance of a TVA+TVA or TVA+OVAI+TVA circuit.

Figure 7 shows the performance measurement method of the TVA+TVA or TVA+OVAI+TVA circuit. Through the function generator, the input signals from 0.04 V_PP_ to 0.1 V_PP_ with 5 cycles at a frequency of 15 MHz were applied to the TVA+TVA or the TVA+OVAI+TVA circuit. A DC signal from the DC supply was applied to the gate and drain of the primary transistor. The DC voltages applied from the DC power supply to the OVAI circuit were 0.5 V, 1 V, and 2 V. A 100:1 and 50-Ω attenuator was used to eliminate amplified high-voltage signals to reduce damage to the oscilloscope [44,45].

**Figure 8:**
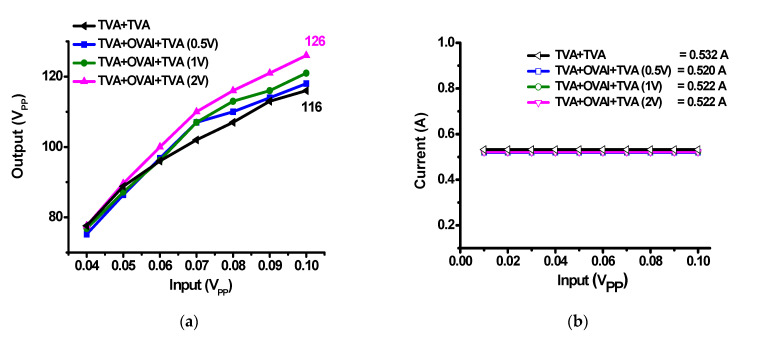
(**a**) Output voltages and (**b**) DC currents of the TVA+TVA, TVA+OVAI+TVA (0.5 V), TVA+OVAI+TVA (1 V), and TVA+OVAI+TVA (2 V) circuits versus input voltages.

Figure 8 shows the measured output voltage and DC current performances versus input voltages when using only the TVA and the TVA with OVAI circuits. The input voltage ranges from 0.04 V_PP_ to 0.1 V_PP_ with a frequency of 15 MHz. The applied DC voltages to the OVAI circuit were 0.5 V, 1 V, and 2 V. Figure 8a shows the output voltages when using only the TVA and the TVA with OVAI circuits. In Figure 8a, TVA+TVA represents the measured output voltages of only the TVA+TVA circuit. TVA+TVA+OVAI (0.5 V), TVA+TVA+OVAI (1 V), and TVA+TVA+OVAI (2 V) represent the measured output voltages of the TVA+OVAI+TVA circuits with the DC voltages of 0.5 V, 1 V, and 2 V, respectively, applied to the OVAI circuit. The TVA+OVAI+TVA circuit has a higher output voltage amplitude than the TVA+TVA circuit. In addition, the amplitude differences between the TVA+TVA and TVA+OVAI+TVA circuits increased as a higher DC voltage was applied to the OVAI circuit. The TVA+OVAI+TVA with a DC voltage of 2 V applied to the OVAI circuit has an output value of 126 V_PP_ at an input voltage of 0.1 V_PP_; hence, its output value is 10 V_PP_ higher than that of the TVA+TVA circuit. This is because the nonlinear capacitance and inductance compensation of the TVA circuits due to the OVAI circuit could increase the output voltage. Figure 8b shows the measured DC current according to different input voltages. The DC current consumptions of the TVA+TVA, TVA+OVAI+TVA (0.5 V), TVA+OVAI+TVA (1 V), and TVA+OVAI+TVA (2 V) circuits were 0.532 A, 0.520 A, 0.522 A, and 0.522 A, respectively. These values are similar to each other because the large internal resistance of the OVAI circuit minimizes the DC current consumption. Table 1 lists the measured values of Figure 8a.

**Figure 9:**
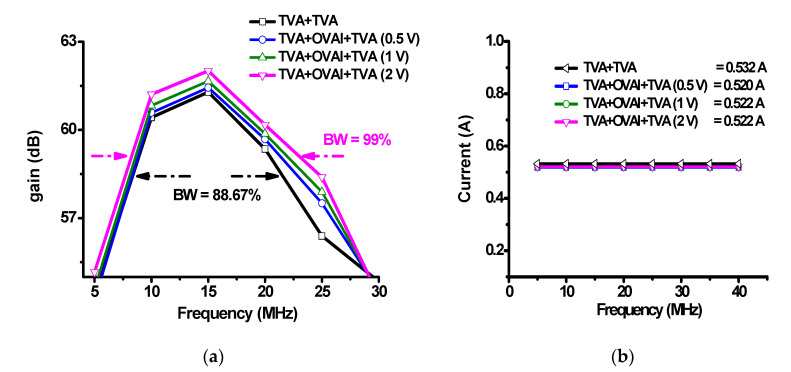
(**a**) Output voltages and (**b**) DC currents of the TVA+TVA, TVA+OVAI+TVA (0.5 V), TVA+OVAI+TVA (1 V), TVA+OVAI+TVA (2 V) circuits versus frequencies.

Figure 9 shows the measured output voltage and DC current performances versus the frequency when using only the TVA and the TVA with OVAI circuits. The frequency ranges from 5 to 40 MHz, with an input voltage of 0.1 V_PP_. As shown in Figure 9a, TVA+TVA represents the measured output voltages of only the TVA+TVA circuit versus frequencies. TVA+TVA+OVAI (0.5 V), TVA+TVA+OVAI (1 V), and TVA+TVA+OVAI (2 V) represent the measured output voltages of the TVA+OVAI+TVA circuits with the DC voltages of 0.5 V, 1 V, and 2 V, respectively, applied to the OVAI versus frequencies. Moreover, TVA+OVAI+TVA (2 V) has a wider bandwidth (99%) than TVA+TVA without OVAI (88.67%) because the OVAI circuit could compensate for the nonlinear effects caused by the output and input capacitances and inductances. Figure 9b shows the measured DC currents of the TVA+TVA, TVA+TVA+OVAI (0.5 V), TVA+TVA+OVAI (1 V), and TVA+TVA+OVAI (2 V) circuits versus the frequency. As shown in Figure 8b, the change in the DC current is small. Table 2 lists the measured values of Figure 9a. The additional OVAI circuit fortunately does not increase the DC current consumption.

As shown in Figure 8a and Figure 9a, the output amplitude and voltage gain with bandwidth are increased as the parasitic input, output, and reverse capacitances (C_ISS_, C_OSS_, and C_RSS_) of the power MOSFET (BSS123) according to the drain voltage of the MOSFET can be dramatically varied. Therefore, we can conclude that the effects of the tuning voltage V_DC_ on the OVAI performances are primarily dependent on the parasitic input, output, and reverse capacitances (C_ISS_, C_OSS_, and C_RSS_) of the power MOSFET (BSS123) in the OVAI circuit.

**Figure 10:**
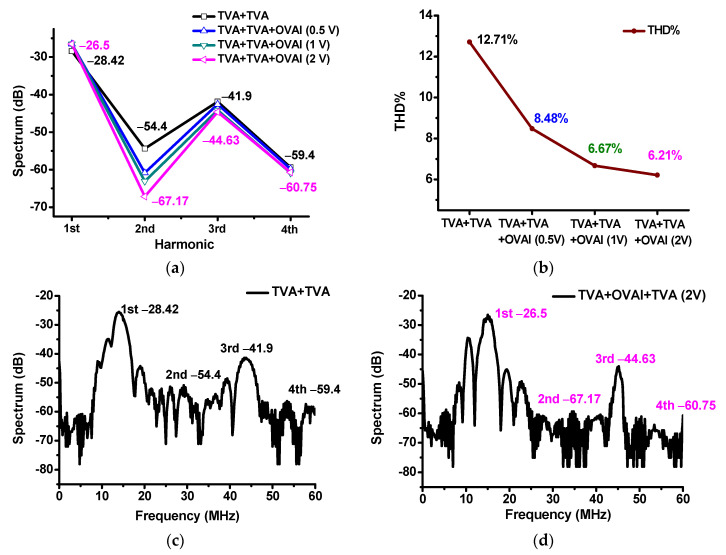
(**a**) Spectrum data versus fundamental and harmonic frequencies; (**b**) THD when using the TVA+TVA, TVA+OVAI+TVA (0.5 V), TVA+OVAI+TVA (1 V), and TVA+OVAI+TVA (2 V) circuits; Spectrum data versus frequency when using the (**c**) TVA+TVA and (**d**) TVA+OVAI+TVA (2 V) circuits.

Figure 10 shows the fast Fourier transform (FFT) and total harmonic distortion (THD) when using the TVA+TVA or TVA+OVAI+TVA circuit. The signal distortions of the developed ultrasound components can be evaluated using the FFT and THD analyses [46,47]. Figure 10a shows the comparison of the harmonic distortion component values when using the TVA+TVA+OVAI (0.5 V), TVA+TVA+OVAI (1 V), and TVA+TVA+OVAI (2 V) circuits. In the THD measurement, the values were measured at the fundamental frequency (1st = 15 MHz), 2nd harmonic (2nd = 30 MHz), 3rd harmonic (3rd = 45 MHz), and 4th harmonic (4th = 60 MHz), and they are expressed as a spectrum (dB). The TVA+TVA and TVA+OVAI+TVA (2 V) circuits had the harmonic distortion components of −28.42 dB and −26.5 dB, respectively, at the fundamental frequency (15 MHz), −54.4 dB and −67.17 dB, respectively, at the second harmonic (30 MHz), −41.9 dB and −44.63 dB, respectively, at the third harmonic (45 MHz), and −59.4 dB and −60.75 dB, respectively, at the fourth harmonic (60 MHz). Figure 10b shows the calculated THD when using the TVA+TVA, TVA+TVA+OVAI (0.5 V), TVA+TVA+OVAI (1 V), and TVA+TVA+OVAI (2 V) circuits. The THD values of the TVA + TVA, TVA+OVAI+TVA (0.5 V), TVA+OVAI+TVA (1 V), and TVA+OVAI+TVA (2 V) circuits were 12.71%, 8.48%, 6.67%, and 6.21%, respectively. Figure 10c,d show the FFTs when using the TVA+TVA and TVA+OVAI+TVA (2 V) circuits. We can confirm that the OVAI circuit helps reduce the second harmonic distortions, thus improving the THD values, because it can compensate for the nonlinear effects caused by the capacitances and inductances of the TVA circuits.

The amplifier performances for the previous and present ultrasound studies are compared in Table 3.

### 3.2. Pulse-Echo Analysis

**Figure 11:**
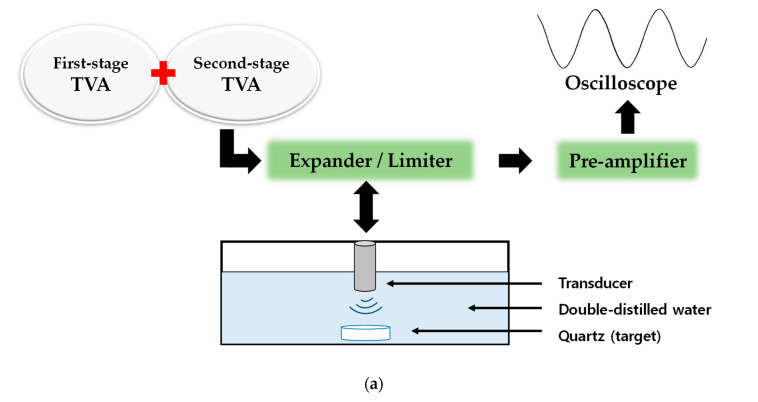

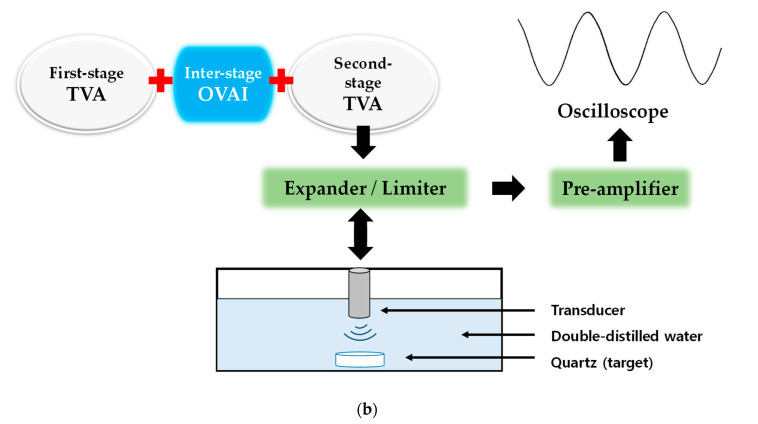
Block diagram of the measurement of the pulse-echo response of the (**a**) TVA+TVA or (**b**) TVA+OVAI+TVA circuit.

The developed ultrasound components need to be tested via pulse-echo analysis [49,50]. In the pulse-echo analysis, the amplitude and pulse width of the echo signal are related to the sensitivity of the transducer, and the −6 dB bandwidth and harmonic distortion components are related to the resolution of the transducer [51,52,53]. Figure 11a,b show the measurement setup for the pulse-echo analysis of the developed TVA+TVA or TVA+OVAI+TVA circuit. The output voltages generated from the TVA+TVA or TVA+OVAI+TVA circuit were passed through an expander circuit to trigger the transducer. The generated acoustic waves were sent back from the quartz, which can reflect the acoustic waves back to the transducer [54,55,56]. The quartz was used to evaluate the performances of the developed OVAI circuit in the pulse-echo analysis because it is a 100% reflector which reflects the acoustic signals from the transducer [57]. Therefore, there is no cavitation observed during the experiment. Before the acoustic waves were sent back to the transducer, the discharged high-voltage waveforms passed through the limiter to the preamplifier [58]. The limiter was used to reduce the discharged high-voltage waveforms to reduce damage to the preamplifier due to the high voltage [59]. Subsequently, the echo signals were passed through the limiter and preamplifier. Therefore, the suppressed discharged high-voltage waveforms are shown, and the echo signal waveforms are displayed on the oscilloscope [60]. 

**Figure 12:**
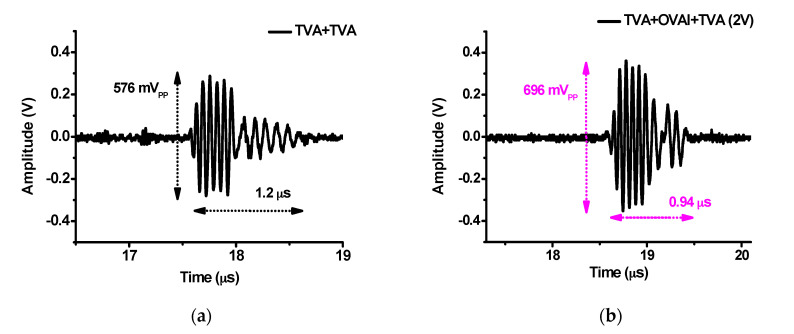
Echo waveforms in the time domain when using the (**a**) TVA+TVA and (**b**) TVA+OVAI+TVA circuits.

Figure 12a,b show the echo amplitude comparison data in the pulse-echo analysis when using the developed TVA+TVA and TVA+OVAI+TVA circuits. The echo output voltage amplitudes when using the developed TVA+TVA and TVA+OVAI+TVA circuits were 576 mV_PP_ and 696 mV_PP_, respectively. Therefore, the echo output voltage amplitude when using the developed TVA+OVAI+TVA circuit was increased by 20.83% due to the increased gain of the amplifier. The echo pulse-widths when using the developed TVA+TVA and TVA+OVAI+TVA circuits were 1.2 µs and 0.94 µs, respectively. Therefore, the echo pulse-width when using the developed TVA+OVAI+TVA circuit was reduced by 21.66%. These results confirm that the developed OVAI circuit helps improve the sensitivity and axial resolution of the transducer because the echo output voltage amplitude and pulse-width are related to the sensitivity and axial resolution of the ultrasound system [61].

**Figure 13:**
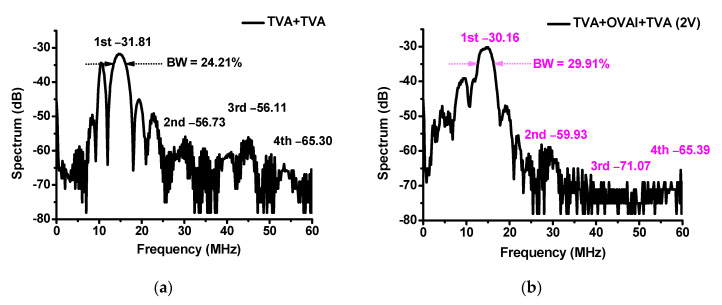
Echo spectrums in the time domain when using the (**a**) TVA+TVA and (**b**) TVA+OVAI+TVA circuits.

Figure 13a,b show the echo spectrum data in the pulse-echo analysis when using the developed TVA+TVA and TVA+OVAI+TVA circuits. The −6 dB bandwidths (BW) when using the developed TVA+TVA and TVA+OVAI+TVA circuits were 29.91% and 24.21%, respectively. Therefore, the developed TVA+TVA circuit does not produce undesirable effects for the bandwidth of the transducer devices. The 2nd, 3rd, and 4th harmonic distortion components when using the developed TVA+TVA circuit were −56.73 dB, −56.11 dB, and −65.11 dB, respectively, whereas the corresponding values when using the developed TVA+OVAI+TVA circuit were −59.93 dB, −71.07 dB, and −65.51 dB, respectively. Therefore, the 2nd and 3rd harmonic distortion components when using the developed TVA+OVAI+TVA circuit were reduced by −3.2 and −14.96 dB, respectively. The calculated THD values when using the developed TVA+TVA and TVA+OVAI+TVA circuits were 3.28% and 1.14%, respectively. These results confirm that the developed OVAI circuit helps reduce the signal distortions of the transducer.

## 4. Conclusions

Mobile ultrasound instruments suffer from low sensitivities caused by piezoelectric transducers under the portable battery. Therefore, several studies of ultrasound devices have been conducted to improve the performances of transducers and circuits. Higher voltage generation from the TVA circuit is highly desirable to increase transducer sensitivity because mobile ultrasound machines have small numbers of TVAs in the multiplexer/de-multiplexer circuits, and small-sized cooling systems have a lower capability to reduce the heat. Currently, commercialized mobile ultrasound machines have heat generation issues and limited transducer channel numbers.

One of the technical solutions to overcome the issues of heat generation and sensitivity is to design a high-performance TVA. However, a multi-stage TVA has problems of limited output voltage generation. Therefore, we proposed a new OVAI circuit to increase the TVA circuit amplitude without affecting the current consumption, minimizing the effects on the impedances of the transducers, because the proposed scheme helps reduce the nonlinear effects of the output impedance of the preceding-stage TVA and the input impedance of the subsequent-stage TVA. The matching filters are located between the TVA circuits, and the transducer and OVAI circuit are located inside the TVA circuits. Therefore, the OVAI circuit could be used to minimize the effect of the matching filters because the filters are used to improve the matching conditions between the TVA circuits and the transducers.

The amplitude and bandwidth when using the developed circuit were higher than those when using only the TVA circuit because the developed circuit helps compensate for the electronic component nonlinearity of the preceding- and subsequent-stage amplifiers. The current consumption when using the developed circuit was similar to that when using only the TVA circuit because the large transistor internal resistance reduces the current consumption. In the pulse-echo mode, the echo signal amplitude and bandwidth when using the developed circuit were higher than those when using only the TVA circuit owing to the increased output voltage amplitude.

Consequently, the increased echo signal amplitude generated from the transducer could improve the transducer sensitivity. This proposed topology is simple to implement with a small size and does not affect the current consumption. Therefore, this scheme helps the ultrasound designer reduce this burden. In addition, increased echo signal quality could lead to the use of larger transducer channel numbers, thus improving the sensitivity and field-of-view of the mobile ultrasound machines. Consequently, they could be beneficial for a clearer and more specific diagnosis of patients.

## Figures and Tables

**Table 1:** Measured output amplitudes in Figure 8a.

Input [V_PP_]	TVA+TVA [V_PP_]	TVA+OVAI +TVA(0.5 V) [V_PP_]	TVA+OVAI +TVA (1 V) [V_PP_]	TVA+OVAI +TVA (2 V) [V_PP_]
0.04	77.6	75.2	76.8	77.6
0.05	88.8	86.4	87.2	89.6
0.06	96	96.8	96.2	100
0.07	102	107	107	110
0.08	107	110	113	116
0.09	113	114	116	121
0.1	116	118	121	126

**Table 2:** Measured output amplitudes in Figure 9a.

Frequency [MHz]	TVA+TVA [V_PP_]	TVA+OVAI +TVA (0.5 V) [V_PP_]	TVA+OVAI +TVA (1 V) [V_PP_]	TVA+OVAI +TVA (2 V) [V_PP_]
5	52.5	51.3	53.7	57.3
10	105	107	110	115
15	116	118	121	126
20	92.8	96.4	98.5	102
25	66	75	78.4	83.1
30	55.2	53.2	52.8	52.1
35	58.4	55	56.2	57
40	59.6	56.3	56.3	56.5

**Table 3:** Comparison data of the amplifier performance enhancement for the previous and present ultrasound studies.

	[19]	[20]	[21]	[48]	Our Work
Class Mode	Class-AB	Class-DE	Class-D	Class-AB	Class-B
Operating Frequency	1 MHz	1.01 MHz	10 kHz	6.5 MHz	15 MHz
Output Voltage	180 V_pp_	-	-	90 V_pp_	126 V_pp_
Output Power	-	0.8 W	2 kW	-	-
HD2	−61.28 dB	-	-	−45 dB	−67.17 dB
HD3	−56.17 dB	−16.4 dB	-	-	−44.63 dB
HD4	-	-	-	-	−60.75 dB
